# How Stable Are Human Aesthetic Preferences Across the Lifespan?

**DOI:** 10.3389/fnhum.2017.00289

**Published:** 2017-05-31

**Authors:** Cameron Pugach, Helmut Leder, Daniel J. Graham

**Affiliations:** ^1^Department of Psychology, John Jay College of Criminal JusticeNew York, NY, United States; ^2^Faculty of Psychology, University of ViennaVienna, Austria; ^3^Department of Psychology, Hobart and William Smith CollegesGeneva, NY, United States

**Keywords:** empirical aesthetics, aesthetic stability, art perception, neuroaesthetics, vision, lifespan development

## Abstract

How stable are human aesthetic preferences, and how does stability change over the lifespan? Here we investigate the stability of aesthetic taste in a cross-sectional study. We employed a simple rank-order preference task using paintings and photographs of faces and landscapes. In each of the four stimulus classes, we find that aesthetic stability generally follows an inverted U-shaped function, with the greatest degree of stability appearing in early to middle adulthood. We propose that one possible interpretation of this result is that it indicates a role for cognitive control (i.e., the ability to adapt cognition to current situations) in the construction of aesthetic taste, since cognitive control performance follows a generally similar trajectory across the lifespan. However, human aesthetic stability is on the whole rather low: even the most stable age groups show ranking changes of at least 1 rank per item over a 2-week span. We discuss possible implications for these findings in terms of existing theories of visual aesthetics and in terms of methodological considerations, though we acknowledge that other interpretations of our results are possible.

## Introduction

How stable are human aesthetic preferences in general, and how does stability vary across the lifespan? The answers to these questions may have a significant impact on our conception of aesthetics. While philosophers (e.g., Hume, 1757;1987) have posited that stability in aesthetic preference in general is a sign of sophistication, and while economists and social scientists have studied population-level stability in preference for economic goods (e.g., Stigler and Becker, 1977) and with regard to political questions (e.g., Druckman and Lupia, 2000), few studies have examined any aspect of stability in aesthetic preference for art, music, or literature over time in individuals (though we review what findings there are below).

Starting with the broader question regarding general levels of aesthetic stability in humans, we note that measures of aesthetic stability bear on both the empirical question of how stable humans tend to be, and also on the methodological question of whether single-trial tests of preference can be considered reliable measures. The latter point echoes similar reevaluations of standard measures of emotion and personality scales, as well as BOLD contrast measures, all of which appear to be reliable at most at a level of 0.7, with typical reliability nearer to 0.5–0.6 (Vul et al., 2009).

With regard to aesthetic stability across the lifespan, folk theories tend to assume that older adults are rather set in their ways. Likewise, parents may observe how a young child’s tastes can become fixed. Adolescents and young adults, on the other hand, are often characterized as inconstant. How accurate are these intuitions?

This picture could be seen to accord with the notion that adulthood is typified by negotiating among competing ecological demands through short-term adaptation, whereas the very young and old need not vary their cognitive set significantly over time since they are protected from great change by the adult population. Yet there may be reasons to expect that these intuitions about the stability of taste could be wrong. An alternative view holds that adulthood is a time when individuals rely on heuristics to determine tastes, just as they do in their habits. The young and the old, on the other hand, may have more freedom to stray from heuristics (though possibly for different reasons).

### Past Work on the Stability of Visual Aesthetics

Past psychophysical findings pertaining to the degree of stability of human visual aesthetic preferences over time have been generated incidentally to the pursuit of different questions; no systematic studies exploring the stability of visual preferences have been performed to our knowledge, and none has examined stability in different age cohorts. We review past findings here.

McManus (1980) examined the long-term stability of preference for rectangles of different ratios and found rather strong consistency of preference in four adult subjects over the course of about 2 years. However, there were some inconsistencies in how the test was administered on the two time-separated trials. McManus et al. (2010) again studied geometric shapes in nine adult subjects and found average individual correlation values over roughly a 5-month span of around 0.6–0.7. Hönekopp (2006) showed a re-test reliability of 0.74 for ratings of face attractiveness over a 1-week span in university students. Park et al. (2010) found that artificially-induced shifts in individual preference within sets of face and landscape stimuli were abolished after a 1-week interval. Finally, Sadacca (1962) found that high school students often showed higher consistency across tasks involving preference and similarity (e.g., for color or verbal material) during a single session than was found for a given task performed twice in a 1-week span. However, this latter study reported motivational problems that may have affected results. Together, these incidental reports of human visual aesthetic preferences do not constitute a systematic estimation of aesthetic stability in the context of lifespan development nor do they necessarily capture what is typical for adult aesthetic preference.

Interestingly, research has shown that people with Alzheimer’s Disease (AD)-related dementia—even those in later stages of the disease—as well as people with frontotemporal dementia (FTD) do not exhibit significantly different levels of stability in aesthetic judgments of many types of paintings and pictures, when these groups of individuals are compared to each other and when they are compared to healthy age-matched control groups (Halpern et al., 2008; Graham et al., 2013; Halpern and O’Connor, 2013). This work has demonstrated the potential for spared cognitive capacities via aesthetic tasks in conditions that typically cause severe and broad cognitive impairment, most notably in semantic memory. Indeed, this research has also shown that patients with AD have substantially worse explicit memory for images compared to controls, as one would expect with a severe disorder like dementia. The lack of significant differences between the groups with dementia and controls in terms of aesthetic stability suggests that explicit memory may not be a major contributor to stability.

### Cognitive Pragmatics and Cognitive Control

What might we expect in terms of typical levels of visual aesthetic stability over the lifespan? Following Craik and Bialystok (2006), who reviewed two major trajectories of cognition throughout the course of the human lifespan, we might expect one of two possible trends. If aesthetic taste exhibits crystallized characteristics—that is, those that involve the accrual of knowledge and experience—our tastes would tend to rapidly become more fixed in early childhood, then increase more slowly but still monotonically into older adulthood, and decline only at the end of life. This outcome could be seen to imply that aesthetic stability is related to representational knowledge, or “cognitive pragmatics” (Craik and Bialystok, 2006). If, on the other hand aesthetic taste is related to cognitive control (i.e., the ability to adapt cognition to current situations) we would expect a trajectory in aesthetic stability with age that matches that of cognitive control. In particular, we would expect a more peaked, unimodal (inverted U-shaped) function. This result could imply that human aesthetics depends fundamentally on our ability to adapt cognitive processes to a given situation, and to maintain heuristics (what we term an “aesthetic construction”) over long periods. This result could be seen as inconsistent with the notion that an individual’s aesthetic preferences depend solely or mostly on the accrual of knowledge and experience. We note that the present study attempts in a general way to determine which of these trajectories is most similar to empirical results for aesthetic stability; however, such potential correspondences do not rule out other explanations for the lifespan trajectory of aesthetic stability.

Here we investigated chronological age-related differences in the stability of visual preference in a cross-sectional study across the lifespan. We collected ranked preference data and memory data for images of painted artwork and photographs depicting the same faces and natural scenes in participants of chronological age 3–99. It should be noted that we are investigating the stability of individuals’ tastes over time, not the idiosyncrasies of individual taste. In previous work, it has been shown that, despite somewhat greater agreement among individuals about landscape images compared to abstract ones, individuals have idiosyncratic tastes whose origins are not well understood (Vessel and Rubin, 2010; Leder et al., 2016). We likewise assume here that tastes vary widely among individuals, but for reasons we do not attempt to discern.

## Materials and Methods

### Overview

**Figure 1:**
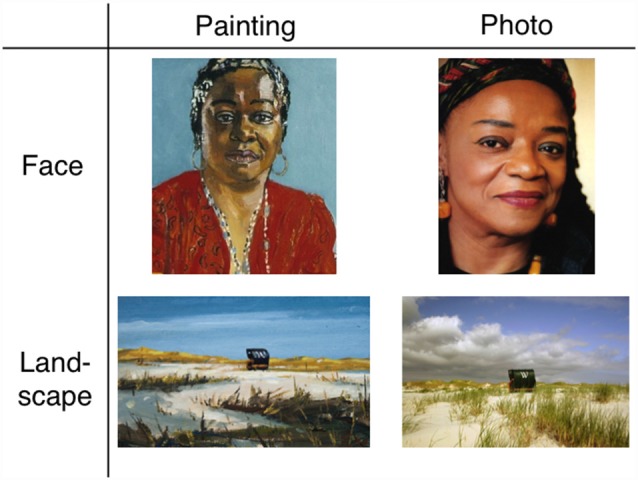
Examples of stimuli used in the experiment. See Supplementary Material for image metadata.

We employed a ranking task and an explicit memory task with image stimuli to test humans across the lifespan. We performed a cross-sectional study of stability in preschool and early elementary school children, adolescents, college-age students and adults. The procedure was the same as in Graham et al. (2013). Participants were asked to rank four sets of eight printed stimuli based on their individual aesthetic preference (see Figure 1 for examples of stimuli, and Supplementary Material for images of all stimuli). Two weeks later, they were asked to repeat the same task. In addition, participants were tested on explicit memory during the second experimental session, prior to the aesthetic stability task. In the explicit memory task, four images in each stimulus set were presented in sequence paired with a distractor image and participants were asked which they had seen before.

The data from the present study were combined with previously collected data for healthy elderly participants in Graham et al. (2013), which were collected using the same images and methodology. All research was conducted in accordance with ethical standards established by the Declaration of Helsinki; written and informed consent was obtained from all participants or their legal guardians, and all phases of the study were approved by the Institutional Review Board at Hobart and William Smith Colleges or the University of Vienna Ethics Committee.

### Participants

#### Young Children

Participants were recruited from four day-care facilities in Geneva, NY, USA: Discovery’s Playground; Roots and Shoots; Geneva Lakefront Childcare Center; and Geneva General Hospital Child Care Center. Children were given permission to participate through written and informed consent of caregivers and through authorization of all programs involved. There were 22 participants (28.9% of total sample; 13 female) age 3–9 (*M* = 6.20, *SD* = 2.08). This group was further subdivided into younger children age 3–6 (*N* = 13, *M* = 4.70) and older children age 7–9 (*N* = 9, *M* = 8.33). There were no incentives given to participants, guardians, or child-care facilities.

#### Adolescents

Participants age 11–16 (14.5% of total sample; 3 female, *N* = 11, *M* = 13.9, *SD* = 2.07) were recruited from the Boys and Girls Club, Geneva, NY, USA. Participants gave verbal assent, and were given permission to participate through written and informed consent of caregivers and through authorization of the Boys and Girls Club. Participants were given a small item (toy sunglasses, lanyard, etc.) as an incentive. There were no incentives given to guardians or the Boys and Girls Club.

#### Undergraduates

Participants age 20–22 (21.1% of total sample; 9 female, *N* = 16, *M* = 21.1, *SD* = 0.62) were recruited from the undergraduate research participant pool at Hobart and William Smith Colleges in return for course credit. All subjects provided written and informed consent.

#### Adults Age 30+

Participants age 30 and older (15.8% of total sample; 8 female, *N* = 12, *M* = 40.8, *SD* = 9.92) were recruited from the campus of Hobart and William Smith Colleges and the Geneva, NY, USA community in return for a small item (mug, thumb-drive, etc.). All subjects provided written and informed consent.

#### Elderly

We reanalyzed data collected in Graham et al. (2013) involving healthy older adults in Vienna, Austria, comprising 15 participants (19.7% of total sample; 10 female) with an average age of 74.2 (*SD* = 13.2). All participants provided written and informed consent and no incentives were given.

### Stimuli

In previous work, Graham et al. (2013) focused on two additional questions pertaining to stimulus level effects with regard to aesthetic stability: (1) Whether handmade (painted) stimuli would produce greater aesthetic stability than other images in the AD group? and (2) What the role of image content, specifically faces, plays in patients with AD? To achieve this, Graham et al. (2013) assembled a stimulus set in the domains of portraiture and landscape painting matched for content with corresponding photographs. Results indicated that only photographs of faces showed significant decreases in stability for the AD group vs. controls, an effect consistent with the possibility of interference from disease-affected face processing systems. Halpern et al. (2008) and Halpern and O’Connor (2013) used three classes of artwork (representational, abstract and quasi-representational) in testing AD and FTD patients, but found only minor stimulus class-related effects. The current work continues the investigation of stimulus-level effects.

The stimuli included four sets of eight images: “painted landscape”, “landscape photo”, “painted portrait” and “portrait photo”. Images were all of recognizable content and were painted in representational styles including Romanticism, Impressionism and Realism. Photographs depicted the same content as the paintings (i.e., same identity as the portrait, or same geographical location as the landscape). Images were culled from Internet sources and books (e.g., Machotka, 1996). See Supplementary Material for images of stimuli.

### Explicit Memory Task

In the explicit memory task, four previously viewed images in each image class (16 total) were paired with distractors in a two-alternative forced-choice task. Participants were asked which image they had seen before. Distractors were painted by the same artist as the target (in the case of painted images), or depicted similar landscapes. Distractor face/portrait images were the same gender and approximate age as those shown in the corresponding target images. See Supplementary Material for images of distractor stimuli.

### Procedure

First, participants ranked the four sets of stimuli. The sets were presented in random order, and the eight stimuli in a given set were arranged on a table in front of the participants in random order. Subjects were asked to create a ranking of the stimuli from “least favorite” to “most favorite”. Participants were told that there was no time limit and that there was no wrong way to rank the stimuli.

Two weeks (14 days) later, participants were given the explicit memory task. Following the memory task, subjects were asked to repeat the rank preference task in the same manner as in the previous session 2 weeks prior.

## Results

In order to calculate how stable an individual’s aesthetic preferences were, we analyzed the per-item numerical change of stimulus rank between session one and session two. The resulting change score ranges from a low score of 0 (no change) to a high score of 4 (each image rank different). This calculation is essentially the *L*_1_ norm (city-block distance) between two sets of rankings made by a given individual for a given task, when considering each set of rankings as a vector in the eight-dimensional orthogonal vector space of stimulus items. The change score was then subtracted from 4 to give an aesthetic stability index, or for simplicity, we call this aesthetic stability. This inversion of the scale serves to represent data in terms of how stable participants are, rather than how unstable they are.

In order to rule out the possibility of random guessing for the preference task, a simulation of 100,000 pairs of random preference rankings was created to measure the average stability value at chance. This value, 1.37, which represents the centroid of the vector space, was compared to the average preference value for each participant group. One-sample *t*-tests confirmed that younger children (*M* = 1.94, *SD* = 0.61), *t*_(12)_ = 3.36, *p* < 0.01, older children (*M* = 2.80, *SD* = 0.40), *t*_(8)_ = 10.67, *p* < 0.001, adolescents (*M =* 2.68, *SD* = 0.40), *t*_(10)_ = 10.83, *p* < 0.001, undergraduates (*M* = 2.93, *SD* = 0.22), *t*_(15)_ = 28.44, *p* < 0.001, adults (*M* = 2.79, *SD* = 0.34), *t*_(11)_ = 14.62, *p* < 0.001, and elderly individuals (*M* = 2.31, *SD* = 0.47), *t*_(14)_ = 7.81, *p* < 0.001, all performed well above chance.

These summary results indicate that on average, even the most stable group—undergraduates—experienced a change in aesthetic preference of at least one rank per item across all image classes, while young children showed on average more than two rank changes per item; see Table 1.

**Table 1:** Descriptive statistics for memory performance and stability across both age group and image category, as well as stability results for each stimulus group.

	N	Mean *(SD)*	Range	Skewness (SE)
Total recall rate (%)	76	0.883 (0.161)	0.67	−1.72 (0.276)
Total stability	76	2.56 (0.539)	2.25	−0.766 (0.276)
Landscape painting stability	75	2.65 (0.687)	3.25	−0.651 (0.277)
Landscape photo stability	75	2.47 (0.819)	3.25	−0.458 (0.277)
Portrait painting stability	73	2.61 (0.672)	3.00	−0.496 (0.281)
Portrait photo stabilty	75	2.58 (0.651)	3.00	−0.836 (0.277)

**Figure 2:**
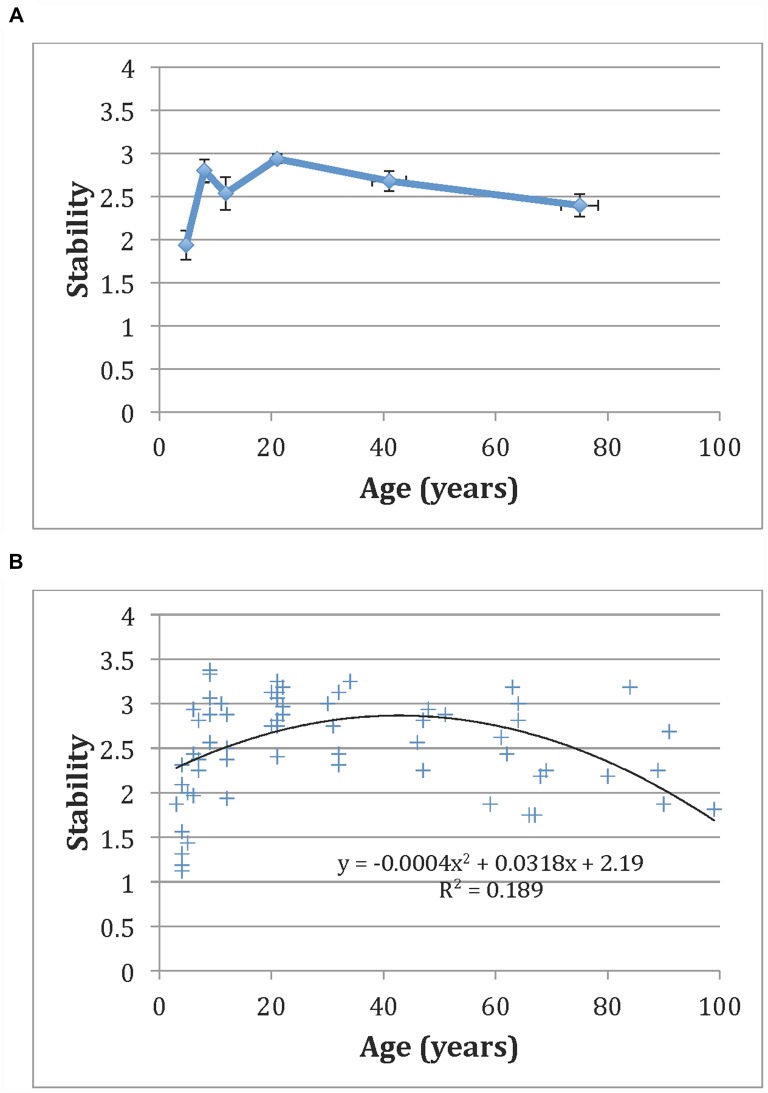
**(A)** Aesthetic stability values as a function of age averaged across stimulus groups plotted by age group.** (B)** The same data for all participants along with associated quadratic fit and fit parameters. Error bars are given in standard error (SE).

Figure 2A shows the overall change score for each of the six participant groups. Differences in stability as a function of age were analyzed using a one-way analysis of variance (ANOVA). Results showed a significant effect of participant group on change score at the *p* < 0.05 level, *F*_(5,70)_ = 10.74, *p* < 0.001. *Post hoc* analyses using the Scheffé criterion for significance indicated that children ages 3–6 (*M* = 1.94 *SD* = 0.61) showed significantly lower stability than children ages 7–9 (*M* = 2.80, *SD* = 0.40), adolescents (*M* = 2.68, *SD* = 0.40), undergraduates (*M* = 2.93, *SD* = 0.22), and adults (*M* = 2.79, *SD* = 0.34). Similarly, elderly individuals (*M* = 2.31, *SD* = 0.47) showed significantly lower stability in comparison to undergraduates, adolescents, or adults (but not to younger or older children). Figure 2B shows all participants’ results as a function of age, along with a fit that demonstrates a significant quadratic relationship (*R^2^* = 0.189, *p* < 0.01). This result suggests an inverted U-shaped relationship with age: aesthetic stability tends to increase with age until early adulthood, where it begins to decreases gradually until the end of life.

**Figure 3:**
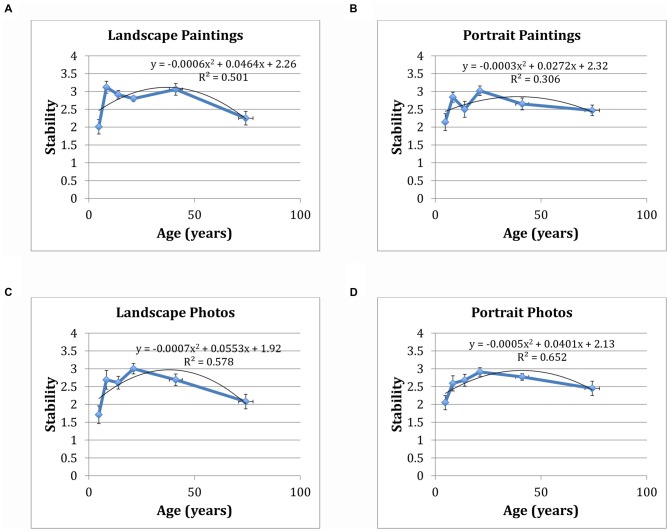
Aesthetic stability values as a function of age group by stimulus group.** (A)** Landscape paintings; **(B)** landscape photos; **(C)** portrait paintings; **(D)** portrait photos. Associated quadratic fits are also shown, along with fit parameters. Error bars are given in SE.

Figure 3 shows mean aesthetic stability values for each participant group as a function of age, organized by image type/category (i.e., landscape paintings, landscape photos, portrait paintings and portrait photos). Stability as a function of each stimulus category was also analyzed using a series of one-way ANOVAs. Of particular interest are the mean differences in each of the six participant groups’ change score when comparing landscape paintings and photos to portrait paintings and photos.

For landscape paintings, results showed a significant effect of participant group on change score, *F*_(5,69)_ = 7.88, *p* < 0.001. *Post hoc* analyses using the Scheffé criterion for significance showed that children ages 3–6 (*M* = 2.01, *SD* = 0.73) were significantly less stable than children ages 7–9 (*M* = 3.12, *SD* = 0.50), adolescents (*M* = 2.91, *SD* = 0.39), undergraduates (*M* = 2.8, *SD* = 0.30) and adults (*M* = 3.06, *SD* = 0.57). Elderly individuals (*M* = 2.25, *SD* = 0.74) were also less stable than children ages 7–9 and adults. Similar effects were also observed for landscape photographs, *F*_(5,69)_ = 5.95, *p* < 0.001. *Post hoc* analyses showed that both children age 3–6 (*M* = 1.71, *SD* = 0.90) and elderly individuals (*M* = 2.08, *SD* = 0.79) were significantly less stable than undergraduate students (*M* = 3.00, *SD* = 0.58).

In comparison, effects of participant group on change score were less prominent for both portrait paintings (*F*_(5,67)_ = 3.03, *p* = 0.02) and portrait photographs (*F*_(5,69)_ = 3.46, *p* = 0.01). *Post hoc* analyses indicated that, for portrait paintings, children ages 3–6 (*M* = 2.14, *SD* = 0.85) were significantly less stable than undergraduates (*M* = 3.02, *SD* = 0.54). Similarly, for portrait photographs, children ages 3–6 (*M* = 2.05, *SD* = 0.72) were significantly less stable than undergraduates (*M* = 2.92, *SD* = 0.48). Thus, fewer mean differences in stability were found across all age groups for portrait paintings and photos in comparison to landscape paintings and photos, though the trajectory of stability of face images as a function of age broadly matches what is observed for landscape images.

An independent samples *t*-test also showed no significant differences in the stability scores of males (*M* = 1.55, *SD* = 0.56) and females (*M* = 1.36, *SD* = 0.51), *t*_(74)_ = 1.54, *p* = 0.13.

### Explicit Memory

Table 2 shows the recall rate (% correct), or explicit memory, for each group of participants. A one-way ANOVA showed a significant effect of participant group on explicit memory, *F*_(5,70)_ = 7.37, *p* < 0.001. *Post hoc* analyses using the Scheffé criterion for significance showed that children ages 3–6 (*M* = 0.76, *SD* = 0.23) recalled significantly less information than children ages 7–9 (*M* = 0.97, *SD* = 0.03), adolescents (*M* = 0.95, *SD* = 0.03), undergraduates (*M* = 0.94, *SD* = 0.11), and adults (*M* = 0.96, *SD* = 0.06). Similarly, elderly individuals (*M* = 0.76, *SD* = 0.17) performed significantly more poorly than older children, adolescents, undergraduates and adults.

**Table 2:** Memory performance (recall rate, %) in each age group (with standard error).

Participant group	Recall rate % (SE)
Younger children	76.2 (6.5)
Older children	97.3 (1.0)
Adolescents	94.9 (0.9)
Undergraduates	93.7 (2.7)
Adults	96.1 (1.6)
Elderly	75.8 (4.2)

Each group’s memory task results were compared to chance performance (0.50) in order to rule out the possibility of random guessing. One-sample *t*-tests showed that younger children (*M* = 0.76, *SD* = 0.23), *t*_(12)_ = 4.01, *p* < 0.01, older children (*M* = 0.97, *SD* = 0.03), *t*_(8)_ = 44.06, *p* < 0.001, adolescents (*M = 0.95*, *SD* = 0.03), *t*_(10)_ = 46.64, *p* < 0.001, undergraduates (*M* = 0.94, *SD* = 0.11), *t*_(15)_ = 16.45, *p* < 0.001, adults (*M* = 0.96, *SD* = 0.06), *t*_(11)_ = 27.47, *p* < 0.001, and elderly individuals (*M* = 0.76, *SD* = 0.17), *t*_(14)_ = 6.1, *p* < 0.001, all performed significantly better than chance.

We note that explicit memory performance in our study should not necessarily be thought of as a correlate of the strength of cognitive pragmatics (crystallized processes) in an individual since the information presented during the experiment was new to the participants.

**Figure 4:**
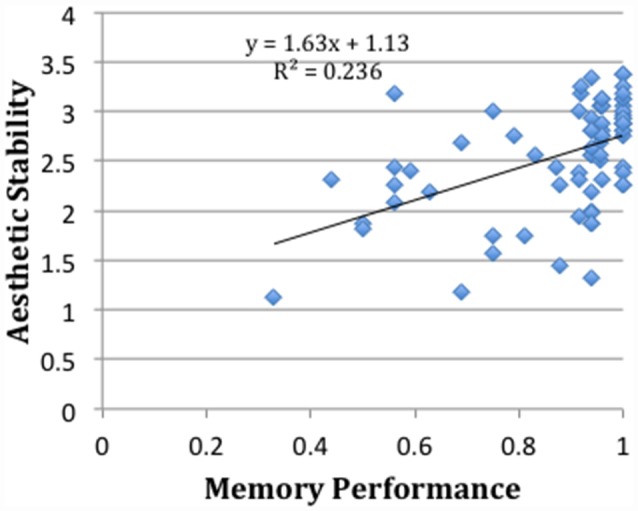
Correlation of memory performance (% correct) and aesthetic stability for each participant, with linear fit and fit parameters.

**Figure 5:**
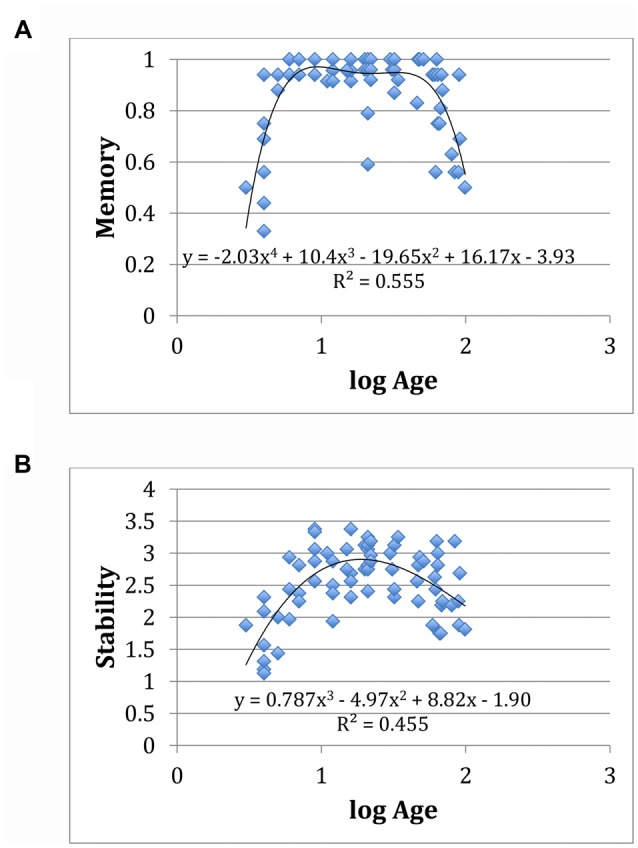
**(A)** Correlation of the logarithm of age vs. memory performance (% correct) fitted with a 4th order (even-symmetric) polynomial. **(B)** Correlation of the logarithm of age vs. aesthetic stability fitted with a 3rd order (odd-symmetric) polynomial.

While aesthetic stability and memory performance are significantly correlated (*p* < 0.01, *R^2^* = 0.236; see Figure 4), further analysis suggests a divergence (though not necessarily a dissociation) between memory performance and aesthetic stability as a function of age. In particular, memory performance as a function of the logarithm of age is well fit by a fourth order (even symmetric) polynomial: the data show a sharp rise in early childhood, a broad plateau through later life, and sharp fall at the end of life. This accords with the notion that cognitive pragmatics remain relatively constant throughout adulthood, declining only towards the end of life when cognitive access (e.g., lexical access, which we note is in part a function of cognitive control) falls off. Aesthetic stability, on the other hand, is well fit as a function of the logarithm of age by a third order (odd symmetric) polynomial that peaks around age 17, with gradual fall off in younger and older people (see Figure 5).

We note also that the correlation between aesthetic stability and memory performance was lower when considering each image class separately. Values of *R*^2^ for landscape paintings, landscape photos, portrait paintings and portrait photos were 0.20 (*p* < 0.001), 0.11 (*p* < 0.01), 0.0005 (*n.s*.), and 0.06 (*p* < 0.05), respectively.

An independent samples *t*-test also revealed that across the entire sample, males (*M* = 0.91, *SD* = 0.13) did not differ from females (*M* = 0.86, *SD* = 0.51) in their explicit memory scores, *t*_(74)_ = 1.48, *p* = 0.14.

### Spearman Correlation Analysis

In order to make our results comparable to other studies of the reliability of experimental measures in psychology, we reanalyzed our full data using a Spearman correlation analysis of rankings in sessions 1 and 2 for all participants. This measure has a simpler scale from 0 (uncorrelated rankings) to 1 (identical rankings) compared to the aesthetic stability index; however, we note that because Spearman is an approximation and aesthetic stability is an exact measure, we chose to use the latter in our main analysis in this article. Moreover, aesthetic stability is readily and intuitively interpretable in terms of rank changes per item.

The grand mean of Spearman ρ values across all participants and stimuli was 0.27. The value was somewhat higher when considering the grand mean of the magnitude of ρ (0.35). However, *p* values in this analysis were only occasionally less than 0.05 (about 13% of all pairs of rankings). Considered along with results from McManus (1980), McManus et al. (2010), and Hönekopp (2006), our results suggest that the reliability of preference judgments falls off rapidly over the scale of days.

### Consistency of Preference for Matched Content

Given that the content of landscape paintings was matched with landscape photographs, and given that the content of portrait paintings was matched with portrait photographs, we also investigated the consistency of each participant in their rankings for these two corresponding stimulus categories.

Data were analyzed using an identical methodology as was used for calculating change scores, where we analyzed each participant’s per item numerical change of stimulus rank between matched landscape paintings and landscape photos, and between matched portrait paintings and portrait photos. Results were calculated for both session one and session two, yielding a total of four scores. As with the change score, a low score of 0 represented no change and a score of 4 indicated each image rank changed maximally. Scores were then subtracted from 4 to represent the degree to which matched landscape stimuli and matched portrait stimuli were ranked consistently (rather than inconsistently).

Results showed that for session 1, the mean consistency score for matched landscape stimuli was 1.99 (*SD* = 0.78) and for matched portrait stimuli was 1.57 (*SD* = 0.63). For session two, the mean consistency score for matched landscape stimuli was 2.19 (*SD* = 0.76) and for matched portrait stimuli was 1.62 (*SD* = 0.77). A paired samples *t*-test comparing consistency scores of matched landscape stimuli across sessions did not yield significant differences (*t*_(72)_ = 1.60, *p* = 0.11). Similarly, a paired samples *t*-test comparing consistency scores of matched portrait stimuli across sessions was not significant (*t*_(71)_ = 0.51, *p* = 0.61).

These results could be viewed in at least two ways: first as a “memory-free” measure of test-retest reliability. From this perspective, our results would appear to confirm that humans are rather inconsistent in their preferences even for tasks with matched content presented in the same session. Alternatively (or in addition), these results could be seen to highlight the highly idiosyncratic nature of aesthetic preference even within individuals.

## Discussion

We find that humans of all ages have relatively little consistency in their aesthetic preferences over a 2-week span, despite performing well above chance in all age groups. Younger adults showed the highest aesthetic stability.

These results indicate that humans on the whole are changeable in their tastes, even over a relatively short time span; inconsistencies among stimuli with matched content reinforce this interpretation.

These findings also agree with past work on non-human primates showing marked inconsistency in preference for visual patterns across short intervals (Rensch, 1957). Thus, we urge caution in the interpretation of studies of aesthetic preference that collect participant rankings or ratings in a single time trial, and we encourage researchers to begin to consider new ways of taking account of these results. Moreover, lower stability values for humans below age 10 and beyond age 65 suggest that additional caution should be exercised in drawing conclusions from single-trial studies in these populations.

On the other hand, previous results showing spared aesthetic stability in people with dementia (Halpern et al., 2008; Graham et al., 2013; Halpern and O’Connor, 2013) suggests that human aesthetics could be more deeply engrained in an individual’s cognition than is implied by the stability of humans in general.

### Lifespan Aesthetic Stability

Contrary to popular stereotypes, our results also indicate that human tastes tend to be most stable in early- to mid-adulthood, and substantially lower in childhood and late adulthood.

We suggest here that our results are consistent with the idea that, at their cognitive root, aesthetic preferences are in part a function of cognitive control, since this trait is maximal in early adulthood, and substantially lower in youth and later adulthood (Craik and Bialystok, 2006).

In other words, adults may display more consistent tastes because they are better able to maintain stable heuristics concerning what they prefer. In terms of the psychology of aesthetics, this would imply that human aesthetics could be a construction that must be maintained over time. As such, one’s accumulated knowledge (i.e., one’s crystallized knowledge, or one’s store of memories and associations)—which peaks in late adulthood, and only declines substantially near the end of life—may play less of a role in aesthetics than is often assumed.

As noted earlier, Sadacca (1962) found that an individual’s characteristic stability appears to emerge in a variety of judgments (preference, similarity) and stimulus types (colors, verbal material). These findings support the notion that changes in stability are broad-based and thus potentially rooted in core cognitive processes. In addition, it suggests that our results may apply to other types of stimuli besides images.

### Implications for Existing Aesthetic Frameworks

Understanding the trajectory of aesthetic stability across the lifespan also helps us develop a better understanding of how aesthetic preferences form, and it provides a new perspective from which to evaluate existing frameworks in the psychology of aesthetics.

One of the most striking conclusions from our results is that it may be difficult to distinguish aesthetic universals from individual differences in aesthetics, since humans overall appear to be rather changeable. Moreover, to the extent that an individual’s aesthetic construction aligns with putative universals, it may be difficult to determine whether this is due to the aesthetic “pull” of universals, or due to the fact that an individual has constructed an aesthetics that aligns with these universals.

If preferences are shaped by a kind of heuristic aesthetic construction that requires a degree of cognitive control in order to be maintained over time, several existing proposals may require reappraisal.

Some (Bullot and Reber, 2013; see also Graham, 2013) argue that substantial historical and critical context is a prerequisite for the aesthetic appreciation of any work of art. Since general representational knowledge of this kind peaks in late adulthood, we might infer that—all else being equal—older adults should be more stable in their preferences compared to younger people, who have less accumulated knowledge. Although we did not measure appreciation in this study, we did find that stability peaks earlier in life, which calls into question the role of representational knowledge in the formation of aesthetic preference.

Other researchers argue that some aspects of visual aesthetics could be innately linked to evolutionary goals related to ecology (see e.g., Vessel and Rubin, 2010; see also Rodway et al., 2016). However, our finding that young children are highly unstable in their preferences for landscapes and landscape paintings complicates the idea that natural scenes show more innate patterns of preference when compared to, for example, abstract art. Indeed, if certain kinds of landscapes are innately preferred, we should see these biases consistently from early childhood onward. Instead, we find that young peoples’ preferences are much more changeable than adult preferences.

Our data show change between childhood and adulthood in the stability of preference for faces and portrait paintings. These results suggest that innate aesthetic biases for face attributes (e.g., for symmetry or averageness) may be relatively weak; this finding is in line with a recent twin study showing far more variation in face preference judgments between monozygotic twins than is expected given the twins’ highly similar performance on measures of face recognition (Germine et al., 2015). These researchers argue that their results suggest environmental effects are at work; that is, each twin could see a different set of faces over the lifespan, which could shape individual taste. But given that identical twins often develop in similar environments, this result may instead be seen as a by-product of the diverse and changeable aesthetic constructions that adults create, which may or may not depend on differential environments.

Some in experimental aesthetics argue that aesthetics—at least with regard to fine art—requires sophisticated problem-solving abilities, for example to extract predictable visual forms in an uncertain visual environment (Van de Cruys and Wagemans, 2011; Muth and Carbon, 2013). But to the extent that humans appreciate this kind of problem solving or pattern-comparison task, this suggestion may be more parsimoniously explained as a kind of aesthetic task that adults are most well suited to. In other words, the apparent aesthetic reward for “figuring out” a painting or musical work may be just one of many aesthetic constructions that adult human viewers—and artists—can create. One may also consider that this kind of aesthetic construction might be particular to university-educated research participants.

### Manipulating Aesthetics

Given our results showing relatively low consistency in taste across time, we are prompted to ask what conditions are necessary for changing human aesthetic response, as has been done in the study of social attitudes and choices (e.g., prospect theory; Kahneman and Tversky, 1979 or Strack and Schwarz, 2007). One avenue for approaching this question in aesthetics is to examine the role of novelty and familiarity. Park et al. (2010) conducted a study involving visual exposure to a variety of stimuli including faces, natural scenes, and geometric figures. They found that the young adult participants preferred familiarity for stimuli involving faces while for stimuli involving natural scenes novelty was preferred. However, these artificially induced shifts in preference were abolished after a 1-week interval, with subjects returning to patterns of preference like those that occurred before the manipulation.

Work by Pelli and Vale (2014) has begun to study manipulations that are capable of changing observer aesthetic response on shorter timescales. However, changing aesthetic preferences over long periods in adults may be difficult to achieve given low “baseline” stability. We therefore encourage further work in this vein.

### Clinical Implications

In terms of geriatric clinical practice, it may be the case that when an individual’s aesthetic taste becomes quite inconsistent across stimulus types or tasks, this could be an indication of deficits or disturbances in neural components underlying cognitive control. Such disturbances might not be uncovered in tests of memory performance or in single-trial tests of cognitive control processes. Aesthetic judgment tasks may also be less burdensome to elders than existing cognitive assessments. However, more work is needed to elucidate potential applications of this kind.

### Caveats

We note that the phased nature of the experiment was such that some participants received small incentives for their participation while others did not. We believe this is at most a minor flaw because the task does not have a single correct response, and participants were well informed about this fact. Indeed, they were not aware that the second session would re-test preference for images from the first session. It remains possible—but unlikely in our view—that participants could suspect that concordant rankings in the two sessions was the desired response.

In addition, we note that the elderly participants were from Austria while participants from other age groups were from the USA. However, both Austria and the USA are considered W.E.I.R.D. (western, educated, industrialized, rich, and democratic; see Henrich et al., 2010) and though there is some work showing systematic differences in aesthetic judgments between Eastern and Western viewers (e.g., Bao et al., 2016), we are not aware of studies showing differences in aesthetic judgments between two W.E.I.R.D. cultures. Nor are we aware of studies showing systematic differences in aesthetic judgments due to socioeconomic status or other demographic factors we did not control for. In any case, because there was no difference in stability between males and females, we believe demographic factors exerted little or no influence on the results.

We also note that it is possible that other measures of aesthetic preference may produce different levels of stability. However, we chose to use rank order preference since this measure could be administered easily to all age groups and is less prone to bias than, for example, ratings. If aesthetic ratings are indeed more stable, this could be due in part to methodological biases in rating studies causing more uniformity in preference, and therefore greater likelihood of stability. In any case, Hönekopp (2006) also found rather low aesthetic stability, as noted above, using ratings of faces over just half the timespan used in the current study. We therefore believe other methodologies for measuring preference will show similar levels of stability as those used here.

### Alternative Interpretations

We acknowledge that alternative interpretations of our data are possible. First, it is possible that stimulus diversity could be a factor. In particular, humans may be more stable for sets of images that are more heterogeneous than those used in the current study. However, diversity is difficult to quantify since it depends on photometric as well as semantic information. In any case, our experiment affords us an opportunity to examine stability for image classes of different relative levels of diversity: in particular, we find essentially the same patterns of stability across age for relatively homogenous image classes (e.g., photographs of faces) and for more heterogeneous classes (landscape photographs and paintings).

It is also possible that our memory data show a ceiling effect, which may have made the correlation between memory and stability somewhat less than it is. However, while our data do not show statistical independence between memory and stability, they do suggest that memory performance alone does not explain the stability results (especially when considered for each stimulus class individually). Moreover, it is known that intact explicit memory is not required for aesthetic stability, as shown in people with dementia (Halpern et al., 2008; Graham et al., 2013; Halpern and O’Connor, 2013). In addition, the fact that stability even for the youngest and oldest participants in our study is well above chance suggests that all participants had at least some fixed (albeit idiosyncratic) criteria for making aesthetic judgments. Future work will aim to test causal links between cognitive control and aesthetic judgment using explicit assessments of both constructs at different age points.

## Conclusion

We found that human taste is rather unstable at all stages of life: aesthetic preferences are quite unstable in early childhood; become increasingly stable in young adulthood; and then gradually become less stable in later adulthood. As such, our results refute the popular impression that young adults are fickle while children and older adults are set in their ways. Our results are consistent with the idea that human aesthetics is rooted in cognitive control, and we propose the notion of an “aesthetic construction”, or a heuristic that guides human aesthetic judgment. While our results do not constitute proof that cognitive control and heuristics underlie aesthetic judgment, they do suggest that this idea warrants further study. We conclude that the study of aesthetic stability provides new perspectives in the fields of empirical aesthetics and neuroaesthetics because it may point to new ways of understanding of aesthetics in terms of core cognitive systems. However, the general instability of human taste over relatively short timespans suggests a need for reevaluation of existing frameworks and methodologies in empirical aesthetics.

## Author Contributions

CP collected data, performed data analysis and assisted in writing the article. HL advised on experiment design, analysis and writing. DJG designed the experiment, performed data analysis and wrote the article.

## Conflict of Interest Statement

The authors declare that the research was conducted in the absence of any commercial or financial relationships that could be construed as a potential conflict of interest.
