# Supplementary material for: How Stable Are Human Aesthetic Preferences Across the Lifespan?
Source: Front Hum Neurosci. 2017 May 31;11:289. doi: 10.3389/fnhum.2017.00289 (PMC5449452; doi:10.3389/fnhum.2017.00289)
Supplement: Supplementary file 1 [file Image_1.pdf]

How stable are human aesthetic preferences across the lifespan?

Cameron Pugach

Department of Psychology
John Jay College of Criminal Justice
New York, NY, USA

Helmut Leder

Faculty of Psychology
University of Vienna
Vienna, Austria

Daniel Graham*

Department of Psychology
Hobart and William Smith Colleges
Geneva, NY, USA
**Corresponding author: graham@hws.edu*
tel.: +1-315-781-4526, fax:+1-315-781-3458

Supplemental Materials

Images of stimuli used in our experiments.

Landscape Paintings (LPa)

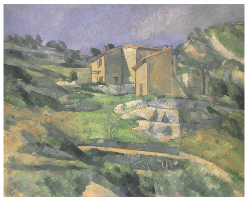
LPa1: Cezanne *"Maison en provence-le vallon"*

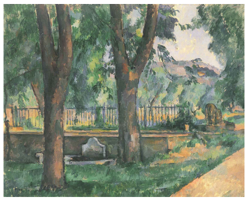
LPa2: Cezanne *"Bassin et lavoir du Jas de Bouffan"*

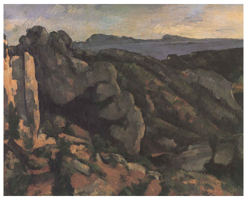
LPa3: Cezanne *"Rochers a l'estaque"*

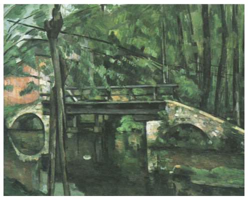
LPa4: Cezanne *"Le Pont de maincy"*

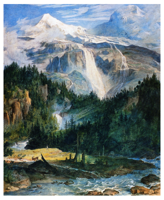

LPa5: Anton Koch *"Der schmadribachfall"*

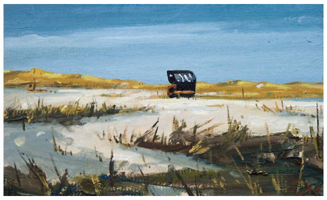

LPa6: Ralf Scherfose *"Dune mit strandkorb"*

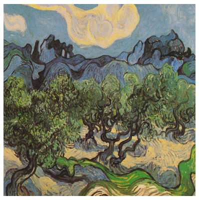

LPa7: Van Gogh *"Olivenbaume"*

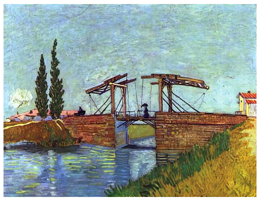

LPa8: Van Gogh *"Die Brucke von Langlois"*

Landscape Photographs (LPh)

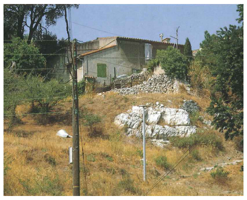

LPh1: Machotka

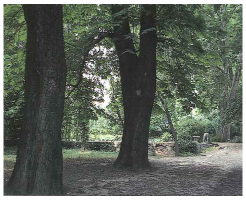

LPh2: Machotka

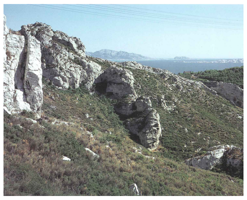

LPh3: Machotka

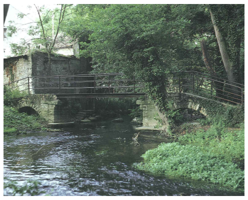

LPh4: Machotka

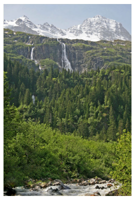

LPh5: *"Der Schmadribachfall"*

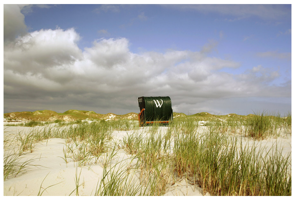

LPh6: *"Amrum"*

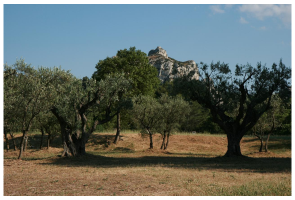
LPh7: *"Olivenbaume in St. Remy"*

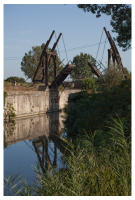
LPh8: *"Brucke von Langlois"*

Portrait Paintings (PPa)

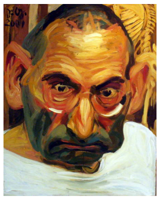
PPa1: Johannes Gruetzke *"Selbstbildnis"*

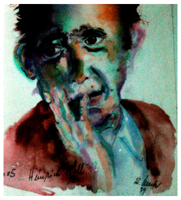
PPa2: Norbert Weck *"portrait Heinrich Boll"*

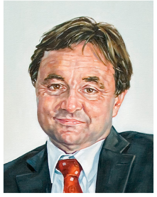
PPa3: Ralf Scherfose *"portrait prof. Dr. Willy Alfred Gutschelhofer"*

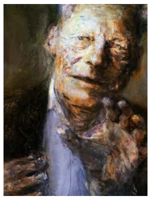
PPa4: Johannes Heisig *"portrait Brandt"*

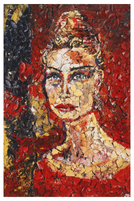
PPa5: Julian Schnabel *"Portrait of Olatz"*

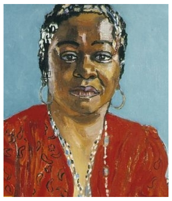
PPa6: Alice Steel *"Faith Ringgold"*

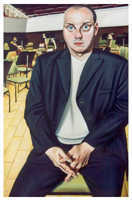
PPa7: Norbert Wagenbrett *"Matthias Goerne"*

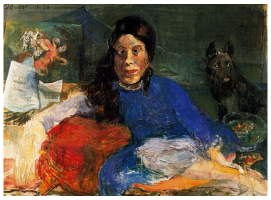
PPa8: Oskar Kokoschka *"Adele Astaire"*

Portrait Photographs (PPh)

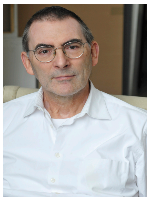
PPh1: *"J. Gruetzke"*

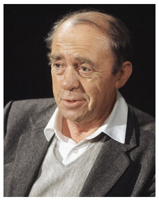
PPh2: *"Heinrich Boll"*

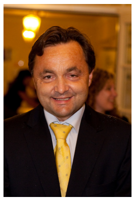
PPh3: *"Gutschelhofer"*

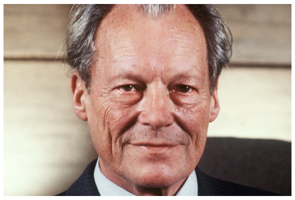
PPh4: *"Willy Brandt"*

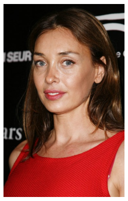
PPh5: *"Olatz Schnabel"*

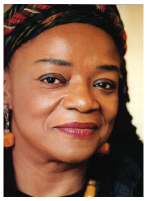
PPh6: *"Faith Ringgold"*

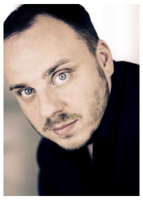
PPh7: *"Matthias Goerne"*

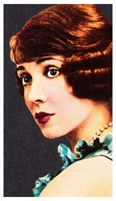
PPh8: *"Adele Astaire"*

Distractor Stimuli (Landscapes)

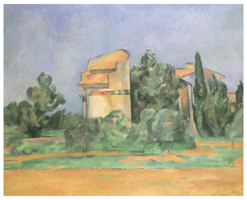
LPa1.1: Cezanne: *"Le Pigeonnier de Bellevue"*

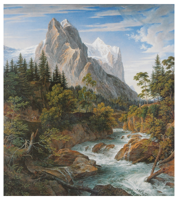
LPa5.1: Anton Koch:
"Reichenbachtal Mit Wetterhorn"

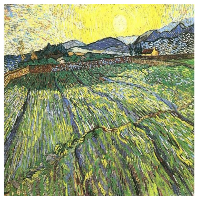
LPa7.1: Van Gogh *"Weizenfelt mit u. Sonne"*

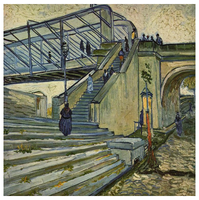
LPa8.1: Van Gogh *"Brucke von Trinquetaille"*

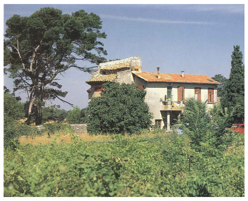
LPh1.1: *"Le Pigeonnier de Bellevue"*

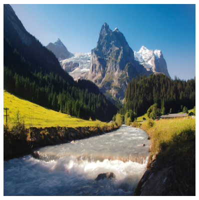
LPh5.1: *"Reichenbachtal Mit Wetterhorn"*

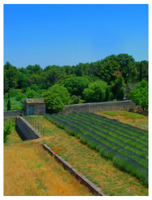
LPh7.1: *"Weizenfelt mit u. Sonne"*

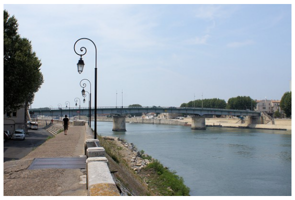
LPh8.1: *"Brucke von Trinquetaille"*

Distractor Stimuli (Portraits)

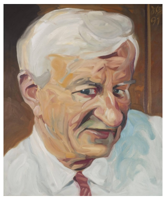
PPa1.1: J. Gruetzke *"Richard von Weizsacker"*

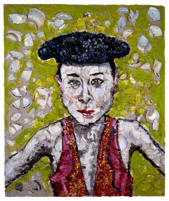
PPa5.1: J. Schnabel *"Portrait Nina Chow"*

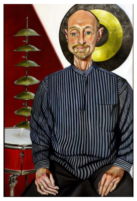
PPa7.1: N. Wagenbrett *"Musiker Wolfram Dix"*

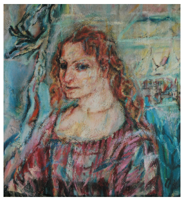
PPa8.1: O Kokoschka *"Alma Mahler"*

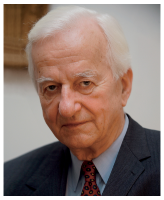

PPh1.1: *"Richard von Weizsacker"*

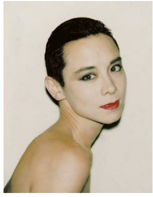

PPh5.1: *"Nina Chow"*

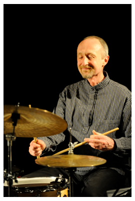

PPh7.1: *"Musiker Wolfram Dix"*

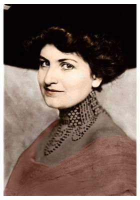

PPh8.1: *"Alma Mahler"*
